# Bacterial Distribution in the Lungs of Children with Protracted Bacterial Bronchitis

**DOI:** 10.1371/journal.pone.0108523

**Published:** 2014-09-26

**Authors:** Ravi Narang, Kelly Bakewell, Jane Peach, Sadie Clayton, Martin Samuels, John Alexander, Warren Lenney, Francis J. Gilchrist

**Affiliations:** 1 Academic Department of Child Health, University Hospital of North Staffordshire, Stoke on Trent, United Kingdom; 2 Paediatric Intensive Care, University Hospital of North Staffordshire, Stoke on Trent, United Kingdom; 3 Institute of Science and Technology in Medicine, Keele University, Keele, United Kingdom; University of Ulster, United Kingdom

## Abstract

**Objectives:**

Flexible bronchoscopy with bronchoalveolar lavage (FB-BAL) is increasingly used for the microbiological confirmation of protracted bacterial bronchitis (PBB) in children with a chronic wet cough. At our centre, when performing FB-BAL for microbiological diagnosis we sample 6 lobes (including lingula) as this is known to increase the rate of culture positive procedures in children with cystic fibrosis. We investigated if this is also the case in children with PBB.

**Methods:**

We undertook a retrospective case note review of 50 children investigated for suspected PBB between May 2011 and November 2013.

**Results:**

The median (IQR) age at bronchoscopy was 2.9 (1.7–4.4) years and the median (IQR) duration of cough was 11 (8.0–14) months. Positive cultures were obtained from 41/50 (82%) and 16 (39%) of these patients isolated ≥2 organisms. The commonest organisms isolated were *Haemophilus influenzae* (25 patients), *Moraxella catarrhalis* (14 patients), *Staphylococcus aureus* (11 patients) and *Streptococcus pneumoniae* (8 patients). If only one lobe had been sampled (as per the European Respiratory Society guidance) 17 different organisms would have been missed in 15 patients, 8 of whom would have had no organism cultured at all. The FB-BAL culture results led to an antibiotic other than co-amoxiclav being prescribed in 17/41 (41%) patients.

**Conclusions:**

Bacterial distribution in the lungs of children with PBB is heterogeneous and organisms may therefore be missed if only one lobe is sampled at FB-BAL. Positive FB-BAL results are useful in children with PBB and can influence treatment.

## Introduction

Chronic cough is a common symptom in children and a frequent reason for specialist referral. [1], [2] Although underlying lung disease must always be excluded, the majority of children with chronic cough have otherwise normal lungs. Protracted bacterial bronchitis (PBB), describes chronic infection of the conducting airways and is characterised by an antibiotic-responsive, wet cough persisting for longer than 4 weeks. [3] Increasing numbers of children are being diagnosed with PBB but it is unclear if this is due to a true increase in incidence or increased recognition. [4] It has been proposed that the incidence of PBB may have been affected by physicians prescribing fewer courses of antibiotics for lower respiratory tract infections that are presumed to be viral [5], [6].

Microbiological confirmation of PBB poses a significant challenge as affected children rarely expectorate sputum. As a result, PBB has the potential to be missed, misdiagnosed or inadequately treated. This can potentially lead to structural damage of the respiratory system and increase the chance of symptoms persisting. [7] The gold standard method of sampling the lower airways in young children is flexible bronchoscopy with bronchoalveloar lavage (FB-BAL). This is safe and has a low rate of complications. [8] At our centre, children with suspected PBB who have not responded to a 2 week course of oral antibiotics are investigated with a chest x-ray (CXR) and FB-BAL. Those with positive BAL cultures are then treated with a 6 week course of an appropriate oral antibiotic. Other centres choose to treat patients with a prolonged course of antibiotics prior to gaining a microbiological diagnosis, reserving FB-BAL for children who do not respond or relapse.

When undertaking FB-BAL in children with suspected PBB, it is our practice to perform single aliquot bronchial washings from each of the 6 lobes (including the lingula). This differs from the European Respiratory Society (ERS) guidance which recommends a triple aliquot sample from a single lobe. [9] Our practice is based on the knowledge that this methodology is safe and has been used to demonstrate a heterogeneous distribution of bacteria in the lungs of children with other respiratory conditions, such as cystic fibrosis (CF). [10] The aim of this study was to review the FB-BAL and CXR results in children with PBB, to assess the bacterial distribution across lung lobes in children with PBB.

## Methods

We retrospectively reviewed the case notes of 50 consecutive children investigated for suspected PBB between May 2011 and November 2013 at the University Hospital of North Staffordshire. As defined by the Health Research Authority guidance this project was service evaluation, patient consent was therefore not obtained. All patient information was anonymised and de-identified prior to analysis. All the FB-BAL procedures had all been performed under general anaesthesia and patients had been requested not to take any antibiotics in the preceding 7 days. The procedure was identical in all patients. The bronchoscope (2.8 mm BF-XP260F or 4.0 mm BF-P260F; Olympus America Inc, Center Valley, USA) was introduced to the lower airway via a laryngeal mask, and the suction port not used until the tip of the bronchoscope was below the level of the carina. Single aliquot bronchial washings were obtained from each lobe by wedging the tip of the bronchoscope into a lobar bronchus and gently instilling 1 ml/kg (maximum 20 ml) of room temperature 0.9% saline under direct vision. The saline had been immediately aspirated into a sterile suction trap. Lobes were sampled in a set order: right upper, right middle, right lower, left upper, lingula and left lower. The samples had been sent to the microbiology laboratory for semi-quantitative bacterial culture. Cytology studies were not performed. Each BAL sample was used to inoculate 5 agar plates: one blood agar, one chocolate agar, one cysteine lactose electrolyte deficient agar, one sabouraud agar and one *Staphylococcus aureus* selective chromogenic agar. The plates were incubated for 48 hours. Visible growth was identified and categorised as: no growth, scanty growth, moderate growth or heavy growth. After each bronchoscopy, the bronchoscope was manually cleaned with detergent/enzymatic solution at the bedside and then reprocessed using an automatic endoscope reprocessor. The bronchoscope was reprocessed before use. Weekly surveillance swabs had been taken from the scopes which were always negative.

## Results

All 50 children had FB-BAL and a CXR. The median (IQR) age of the children was 2.9 (1.7–4.4) years and the median (IQR) duration of their cough was 11.0 (8.0–14.0) months. Positive cultures were obtained from 41/50 (82%) of children; the total number of positive cultures was 64. Of those with positive cultures, 25 children (61%) grew 1 organism, 9 (22%) grew 2 different organisms and 7 (17%) grew 3 or more different organisms. The most commonly cultured organisms were: *Haemophilus influenza* (25 children), *Moraxella catarrhalis* (14 children), *Staphylococcal aureus* (11 children), and *Streptococcus pneumonia* (8 children). There was no relationship between the number of lobes with a positive culture and the culture quantity. Only 15 (30%) of the CXR were reported as normal. The most common abnormalities were bronchial wall thickening in 24 children (48%), increased bronchial markings in 10 (20%) and consolidation in 7 (14%).

If we had only sampled the most affected or the RML (as per the ERS guidance), 17 organisms (28 positive cultures) would have been missed in 15 children. None of these 15 children had a most affected lobe identified radiologically or macroscopically during the FB-BAL procedure. The RML result was therefore used. Eight of the 15 children had no organisms cultured from the RML. Only sampling a single lobe would therefore have led to 7 (14%) children having incomplete microbiological information and 8 (16%) having a false negative bronchoscopy result. If we had limited sampling to the lingula and the RML then 11 organisms (13 positive cultures) would have been missed in 10 (20%) patients, 6 (12%) of whom would have had no organisms cultured at all. See Table 1.

**Table 1:** FB-BAL results from the 15 patients in whom positive cultures would have been missed if the right middle lobe had been sampled alone.

	RUL	RML	RLL	LUL	Lingula	LLL
1	HI, MC			HI		
2		HI, MC	HI, MC	HI, MC	HI, MC, SP	HI, MC, SP
3	HI, MC, SP	HI, MC, SP	HI, MC, SA	HI, MC, SP	HI, MC, SP	HI, MC, SP
4						HI
5			SA	SA	SA	SA
6	HI, MC, CA	HI, CA	HI, CA	HI, MC, CA	HI, CA	HI
7				SA		SA
8	SA	HI, SA	HI, SA, HP	HI, SA		SA
9						MC
10		HI, SP	HI, SP, SA	HI	HI, SP	HI, SP
11						GNB
12				MC	MC	MC
13						HI
14		HP	HI		HI	HI
15	MC, SP	MC	SA		SA	MC

RUL: right upper lobe, RML: right middle lobe, RLL: right lower lobe, LUL: left upper lobe, LLL: left lower lobe, CA: *Candida albicans,* GNB: Gram-negative bacillus, HI: *Haemophilus influenza,* HP: *Haemophilus parainfluenzae,* MC: *Moraxella catarrhalis,* SA, *Staphylococcal aureus*; SP, *Streptococcus pneumoniae*.

All 41 patients with a positive BAL culture were treated with a 6 week course of oral antibiotics. The most commonly prescribed antibiotics were co-amoxiclav (n = 24, 59%), clarithromycin (n = 5, 12%), amoxicillin (n = 4, 10%) and flucloxacillin (n = 4, 10%). Two of the organisms had in-vitro resistance to co-amoxiclav and 11 patients’ isolated *Staphylococcus aureus* which our microbiology department recommends treatment with flucloxacillin or clarithromycin rather that co-amoxiclav.

## Discussion

FB-BAL with the sampling of 6 lobes (including the lingula) has previously demonstrated a heterogeneous distribution of bacteria within the lungs of children with CF. [10] The data from the current study suggest that there is similar heterogeneity in the bacterial distribution in PBB. Due to this heterogeneity, if we had limited our sampling to one or two lobes a number of organisms would have been missed. This would have resulted in incomplete microbiological data or false negative results and ultimately the wrong antibiotic being prescribed or antibiotics being withheld completely. We have demonstrated that positive cultures were obtained from 41/50 (82%) of FB-BAL procedures. This is higher than the rates of 46% and 63% seen in previous studies in which only a single lobe was sampled. [11], [12] Our higher rate of positive cultures seems to be related to the sampling of 6 lobes as if we had only sampled one lobe (RML) the percentage of culture positive procedures would have fallen to 33/50 (66%) and if we had sampled 2 lobes (RML and lingula) it would have been 35/50 (70%).

When performing a triple aliquot BAL, it is recommended that the 1^st^ aliquot is sent for microbiological culture as this is a more proximal sample. [9] The 2^nd^ and 3^rd^ aliquots are pooled for cytology and non-cellular studies as they are more distal samples. When undertaking single aliquot bronchial washings it is not therefore appropriate to send cytology studies. If BAL cytology is undertaken in patients with PBB it demonstrates elevated neutrophil counts. [3] In some clinical situations this could be used to confirm that a positive BAL culture is caused by infection and not colonisation but as all patients with PBB are symptomatic, no positive airway culture could be presumed to be due to colonisation. This means that cytology results are not included as a diagnostic criterion for PBB and we do not think they would have provided any additional information in this particular clinical setting.

The mainstay of PBB treatment is oral antibiotics, although the use of intravenous and nebulised antibiotics has been reported. [11] If there has been microbiological confirmation of PBB then the antibiotics can be chosen according to the isolated organism and its sensitivities. If treatment is being started “blind” then co-amoxiclav is the most widely used first-line treatment. [13] When this cannot be used, a macrolide or cephalosporin are other options. [14] In our study, although co-amoxiclav would have been an appropriate antibiotic for most of the children, the bronchoscopy results led us to using a different antibiotic in 17. For 4 children the cultures enabled us to prescribe amoxicillin rather than co-amoxiclav. Two isolated organisms with in-vitro resistance to co-amoxiclav and 11 children isolated *Staphylococcus aureus* which our microbiology department recommends treatment with flucloxacillin or clarithromycin rather than co-amoxiclav.

In summary, this study confirms that FB-BAL is a useful investigation in children with PBB and the results can influence management. It also suggests that when undertaking FB-BAL in such children, a number of organisms may be missed if sampling is limited to one or two lobes. Further studies are required to evaluate the most appropriate timing of bronchoscopy in children with PBB and the optimal duration of treatment.
